# Potassium Ascorbate with Ribose: Promising Therapeutic Approach for Melanoma Treatment

**DOI:** 10.1155/2017/4256519

**Published:** 2017-09-24

**Authors:** Carlotta Cavicchio, Mascia Benedusi, Erika Pambianchi, Alessandra Pecorelli, Franco Cervellati, Vinno Savelli, Duccio Calamandrei, Emilia Maellaro, Giorgio Rispoli, Emanuela Maioli, Giuseppe Valacchi

**Affiliations:** ^1^Department of Life Sciences and Biotechnology, University of Ferrara, Ferrara, Italy; ^2^Plants for Human Health Institute, Department of Animal Sciences, North Carolina State University, Kannapolis, NC, USA; ^3^Department of Medicine, Surgery and Neurosciences, University of Siena, Siena, Italy; ^4^Department of Biotechnology, Chemistry and Pharmacy, University of Siena, Siena, Italy; ^5^Department of Molecular and Developmental Medicine, University of Siena, Siena, Italy; ^6^Department of Life Sciences, University of Siena, Siena, Italy

## Abstract

While surgery is the definitive treatment for early-stage melanoma, the current therapies against advanced melanoma do not yet provide an effective, long-lasting control of the lesions and a satisfactory impact on patient survival. Thus, research is also focused on novel treatments that could potentiate the current therapies. In the present study, we evaluated the effect of potassium ascorbate with ribose (PAR) treatment on the human melanoma cell line, A375, in 2D and 3D models. In the 2D model, in line with the current literature, the pharmacological treatment with PAR decreased cell proliferation and viability. In addition, an increase in Connexin 43 mRNA and protein was observed. This novel finding was confirmed in PAR-treated melanoma cells cultured in 3D, where an increase in functional gap junctions and a higher spheroid compactness were observed. Moreover, in the 3D model, a remarkable decrease in the size and volume of spheroids was observed, further supporting the treatment efficacy observed in the 2D model. In conclusion, our results suggest that PAR could be used as a safe adjuvant approach in support to conventional therapies for the treatment of melanoma.

## 1. Introduction

Cutaneous melanoma is the most aggressive form of skin cancer representing over 10% of all skin cancers but is responsible for more than 80% of skin cancer-related deaths [1]. In addition, its incidence is growing and has even doubled in the last 10 years: it has been estimated that, in the next future, it will be the fifth most common cancer in American men and the seventh most common cancer in American women, accounting for 5% and 4% of all new cancer cases, respectively [2].

Many risk factors for melanoma have been identified, including environmental and genetic factors, most likely acting in combination. Among endogenous factors, the most relevant are mutations in *BRAF* (mainly the specific mutation V600E), which are observed in ~60% of patients with nonfamilial, cutaneous melanomas [3], and the presence of a large number of nevi and skin phenotype 1 or 2 (fair skin, hair, and iris) [4]. Among exogenous causes, increased risk of melanoma has been associated with overexposure to natural or artificial UV radiation [5, 6].

Regarding the treatment of melanoma, the surgical removal is still the cornerstone of treatment in the early stages of the tumor. For advanced or metastatic melanoma, depending on tumor spread, affected organs, and the patient's general health, several systemic therapies can be chosen, including cytotoxic agents (also combined to radiotherapy) and, more recently emerged, immune-checkpoint blockers or molecular targeted inhibitors [7].

Among adjuvant therapies, IFN-*α* is the only approved treatment for melanoma [8]. Because of the significant side effects of IFN-*α* (e.g., nausea, fatigue, and neutropenia) [9, 10], and the short-lived response to this treatment, research is focused on novel or reappraised adjuvant therapies in support to the conventional ones. On this subject, a growing body of literature has investigated the efficacy of PAR, a compound formed by potassium bicarbonate (KHCO_3_), L-ascorbic acid (AA), and D-ribose (D-Rib). PAR has been reported to have anticancer effects *in vitro* [11, 12] as well as *in vivo*, for example, in precarcinogenic conditions such as genetic syndromes (Beckwith-Wiedemann, Prader-Willi, and Costello Syndromes), which are characterized by an increased risk of malignancies and neoplasms. Interestingly, after once-a-day continuous treatment with PAR, a few patients with these syndromes were monitored for 9–30 months and an improvement of their clinical conditions was observed; most importantly, none of them developed tumors in the follow-up period of ten years [13–15]. PAR has also given encouraging results when used in neoplastic patients undergoing radio- and chemotherapy, increasing survival from five to ten years [12, 16], and in patients with mesothelioma and prostate cancer [17–19].

It is thought that reduction of neoplastic risk afforded by PAR is allowed by different mechanisms; these manifold actions are given by the individual substances, which seem to have additive or synergistic effects [20]. In particular, AA, at pharmacological doses, has shown antiproliferative, antimetastatic [21], antiangiogenic [11], and immunostimulatory properties [22]; KHCO_3_ restores intracellular levels of K+, which are deeply decreased in most cancer cells; and ribose contributes to correct the hypokalemic condition behaving as a catalyst [23].

Taken together, the data from the literature suggest that PAR could be useful as a new adjuvant treatment against cancer. In addition, skin tissues offer a peculiar way to act, which is the topical application that allows the administration of relatively high drug concentration and with minimum significant metabolic transformation.

Thus, the aim of our study was to investigate the effect of PAR on cell proliferation and cell-to-cell communication in human melanoma cells.

## 2. Materials and Methods

### 2.1. Cell Culture

A375 melanoma cells (from ATCC) were grown in Dulbecco's modified Eagle's medium (DMEM, Lonza, Milan, Italy) supplemented with 10% fetal bovine serum (FBS, EuroClone, Milan, Italy), 1% of L-glutamine (Lonza, Milan, Italy), and 1% of penicillin/streptomycin antibiotics (Lonza, Milan, Italy). The cells were maintained at 37°C in a humidified 5% CO_2_ atmosphere. A375 cells have BRAF (V600E) and p16 mutations.

### 2.2. Potassium Ascorbate with Ribose (PAR) Treatment

In preliminary experiments performed in 2D model, cells were treated with a wide range of concentrations of PAR (from 100 *μ*M to 10 mM). In all subsequent experiments, the concentration range was restricted to 500 *μ*M and 2 mM, which proved to be the lowest effective doses (for convenience, the concentrations are referred to ascorbic acid). The mixture was prepared by dissolving potassium bicarbonate, ascorbic acid, and ribose powders in culture medium in the dark (because they are light-sensitive), using nonmetallic spatulas (to avoid oxidation of ascorbic acid).

### 2.3. Cytotoxicity Assay

After 24 hrs of PAR treatments at the concentration range from 100 *μ*M to 2 mM, cell culture media were collected, and cytotoxicity was evaluated as release of LDH (lactate dehydrogenase) in the medium, according to manufacturer's instructions (EuroClone, Milan, Italy) as previously described [24].

### 2.4. Cell Proliferation

5-Bromo-2′-deoxyuridine (BrdU) assay was applied to detect the proliferation rate of A375 cells; the BrdU procedure was carried out according to the manufacturer's instructions (BrdU test kit, Roche, Milan, Italy) as previously described [25].

### 2.5. Quantitative Real-Time PCR

Quantitative real-time PCR was carried out as described below; briefly, total RNA was extracted, using TRIzol reagent (Invitrogen, Carlsbad, CA, USA), from 2 × 10^5^ A375 cells for each experimental condition, according to the manufacturer's recommended procedure. The purity and amount of isolated RNA were analyzed using Nanodrop-ND 1000 (Thermo Fisher Scientific Inc., Wilmington, USA).

First-strand cDNA was generated from 1 *μ*g of total RNA using the iScript cDNA Synthesis Kit (Bio-Rad, Milan, Italy). The primer pairs for the gene of interest and housekeeping genes were obtained from the Real-Time PCR GenBank Primer and Probe Database Primer Bank, RTPrimerDB (Table 1).

Quantitative real-time PCR (qPCR) was performed using SYBR green on the CFX Multicolor real-time PCR detection system (Bio-Rad, Milan, Italy). The final reaction mixture contained 300 nM of each primer, 1 *μ*l of cDNA, and 7 *μ*l of iQ SYBR Green Supermix (Bio-Rad, Milan, Italy). RNase-free water was used to bring the reaction mixture volume to 15 *μ*l. All reactions were performed in triplicate. Real-time PCR was initiated with a 3 min hot-start denaturation step at 95°C and then performed for 40 cycles at 95°C for 3 s and 60°C for 5 s. During the reaction, fluorescence, and therefore the quantity of PCR products, was continuously monitored by Bio-Rad CFX Manager software (Bio-Rad, Milan, Italy). Primers were initially used to generate a standard curve over a large dynamic range of starting cDNA quantities, permitting calculation of the amplification efficiency (a critical value for the correct quantification of expression data) for each of the primer pairs. Ribosomal proteins L13a (RPL13a) and L11a (RPL11a) and GAPDH were employed as housekeeping genes. Samples were compared using the relative cycle threshold (CT). After normalization to more stable mRNA RPL13a, RPL11a, and GAPDH, the fold increase or decrease was determined with respect to control, using the formula 2^−ΔΔCT^, where ΔCT is (gene of interest CT) (reference gene CT), and ΔΔCT is (ΔCT experimental) (ΔCT control).

### 2.6. Protein Extraction and Quantification

Cells were seeded in six-well cell culture plates (1 × 10^6^ cells/ml). After treatments, cells were washed with ice-cold PBS and lysed in ice-cold lysis buffer (20 mM Tris pH 8, 150 mM NaCl, 1% Triton X-100, 1 mM sodium orthovanadate, 1 *μ*g/ml leupeptin, 1 *μ*g/ml aprotinin, 1 *μ*g/ml pepstatin, 10 *μ*g/ml phenylmethylsulfonyl fluoride (PMSF), and 5 mM *β*-glycerophosphate) (Sigma, Milan, Italy). After centrifugation (15,000 ×g, 15 min at 4°C), the supernatants were collected. Protein concentration was determined by Bradford method (Bio-Rad protein assay; Bio-Rad, Milan, Italy).

### 2.7. Western Blot Analysis

60 *μ*g of boiled proteins was resolved on 10% sodium dodecyl sulphate-polyacrylamide electrophoresis gels. Gels were electroblotted onto nitrocellulose membranes and then blocked for 1 hr in Tris-buffered saline, pH 7.5, containing 0.5% Tween 20 and 5% milk. Membranes were incubated overnight at 4°C with the appropriate primary antibody: anti-Connexin 43, diluted 1 : 1000 (Santa Cruz Biotechnology Inc., Santa Cruz, CA, USA); monoclonal anti-*β*-actin-peroxidase antibody, diluted 1 : 25,000 (Sigma-Aldrich, Saint Louis, MO, USA). The membranes were then incubated with horseradish peroxidase-conjugated secondary anti-Rabbit antibody (1 : 10,000) for 1 hour, and the bound antibodies were detected by chemiluminescence (Bio-Rad, Milan, Italy). *β*-Actin was used as loading control. The blots were first probed for the Connexin 43 and then stripped and reprobed with *β*-actin as the loading control.

Images of the bands were digitized and the densitometry analysis was performed using ImageJ software.

### 2.8. Generation and Treatment of Melanoma Spheroids

A375 cells were initially treated for 24 hrs in a 2D culture. Then, they were detached and melanoma 3D spheroids were generated by a hanging drop technique: drops of 27 *μ*l, each containing 1 × 10^4^ cells, were placed on the lid of a nonadhesive petri dish, containing 3 ml of PBS. Spheroids were incubated for 28 days at 37°C in a 5% CO_2_ atmosphere. 10 *μ*l of medium with or without PAR were added per drop once a week.

### 2.9. Electrophysiological Analysis

Cell recording was performed at RT under visual control, using the whole-cell configuration of the patch-clamp technique. Lucifer yellow (LY), a fluorescent dye (350 *μ*M concentration, excitation: 425 nm, emission: 528 nm), was injected through the patch pipette in a spheroid cell, and fluorescence images of untreated and treated cells (500 *μ*M and 2 mM PAR) were acquired concurrently to the whole-cell recording. The current amplitude (recorded employing an Axopatch 200B; Molecular Devices, Sunnyvale, CA, USA) elicited by repetitive −10 mV pulses was used to measure the seal resistance during cell-attached recording. Once the whole-cell recording configuration was obtained, the current transients produced by these repetitive pulses were used to measure the cell membrane resistance (Rm), the access resistance (Ra), and the membrane capacitance (Cm).

The stability of the recording was checked by routinely measuring Rm, Ra, and Cm. Patch pipettes were filled with an intracellular solution containing the following: pipette solution (mM): KCl 140, MgCl_2_ 1, EGTA 5, HEPES 10, Mg-ATP 5, lucifer yellow 0.5%, pH 7.2; external solution (nM): NaCl 150, KCl 4, MgCl_2_ 1, CaCl_2_ 1.2, HEPES 10, Glucose 10, pH 7.4. All chemicals were purchased from Sigma (St. Louis, MO, USA). Recordings were filtered at 2 kHz via an eight-pole Butterworth filter (VBF/8 Kemo, Beckenham, UK), sampled on-line at 5 kHz by a Digidata 1322A (Molecular Devices, Sunnyvale, CA, USA) connected to the SCSI port of a Pentium computer running the pClamp 9.0 software package (Molecular Devices, Sunnyvale, CA, USA), and stored on disk. Data were further low-pass filtered offline at 200 or 500 Hz using a Gaussian filter, or by using the “running average” routine of SigmaPlot (version 8.0; Jandel Scientific, San Rafael, CA, USA), and analyzed using Clampfit (version 9.0; Molecular Devices, Sunnyvale, CA, USA).

### 2.10. Fluorescence Imaging

Besides bright-field viewing of the cells, the camera and its controlling software (AquaCosmos, version 2.5.3.0; Hamamatsu Photonics, Tokyo, Japan) were also employed for fluorescence imaging analysis, in order to assess the presence of gap junctions by measuring the diffusion of lucifer yellow in the spheroid (CH, dilithium salt; dissolved in intracellular solution at 350 *μ*M concentration; excitation: 425 nm, emission: 528 nm). The excitation light was generated by a monochromator (Polychrome II, Till Photonics, FEI, Hillsboro, Oregon, USA) coupled to the epifluorescence port of the microscope via an optical fiber. Image analysis was performed by using AquaCosmos software.

### 2.11. Dimensional Evaluation of Spheroids

**Figure 1:**
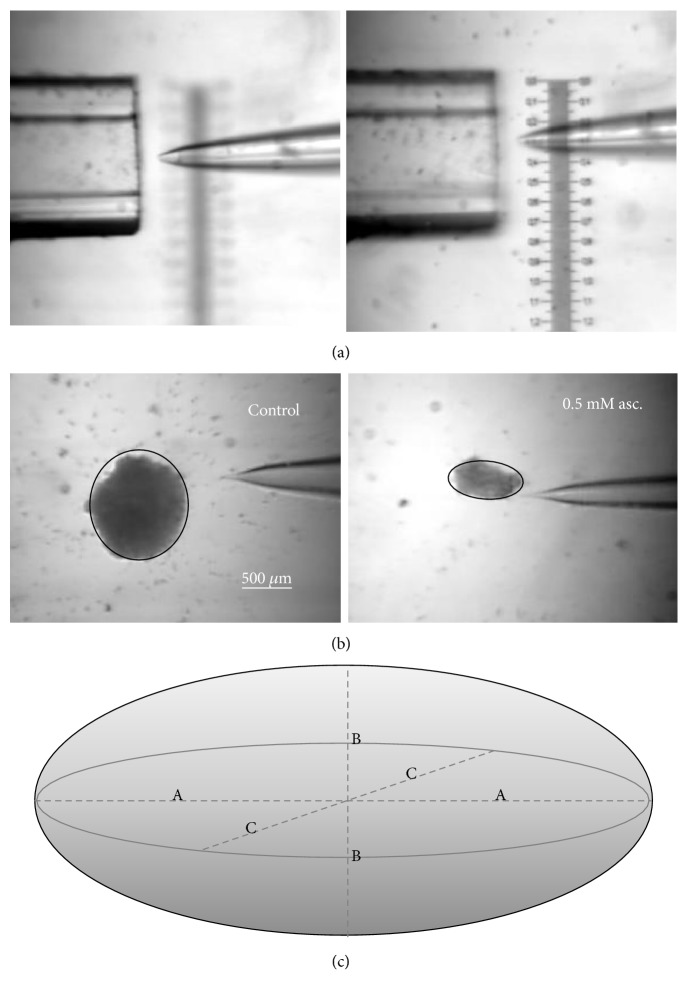
Description of method used to measure the sizes of the melanoma spheroids. (a) Calibration of the Vernier scale. Magnification 40x. (b, c) Ellipsoid dimensions.

The length and width of a spheroid was established with a graduated slide (Figure 1(a), right panel). The depth was assessed by measuring the vertical movement of the tip of a patch pipette with a micromanipulator whose knob was equipped with a Vernier scale (or nonius). This scale was calibrated with square glass tubes of different sizes, by bringing the pipette tip at the same focus of the square tube top (Figure 1(a), left) and bottom (Figure 1(a), right), while viewing the field with a high magnification stereomicroscope equipped with a digital camera.

The depth of a spheroid was evaluated by measuring the travel of the micromanipulator knob when the pipette was focused at the top and then at the bottom of the spheroid. Finally, the spheroid shape was interpolated with an ellipsoid (Figures 1(b) and 1(c)). The spheroid volume V was calculated with the equation *V* = 4/3*πabc*.

### 2.12. Statistical Analysis

For each of the variables tested, two-way analysis of variance (ANOVA) was used. A significant result was indicated by a *p* value < 0.05. Data are expressed as mean ± SD of triplicate determinations obtained in 3 independent experiments.

## 3. Results

### 3.1. Cytotoxic Effect of PAR on Human Melanoma Cells

**Figure 2:**
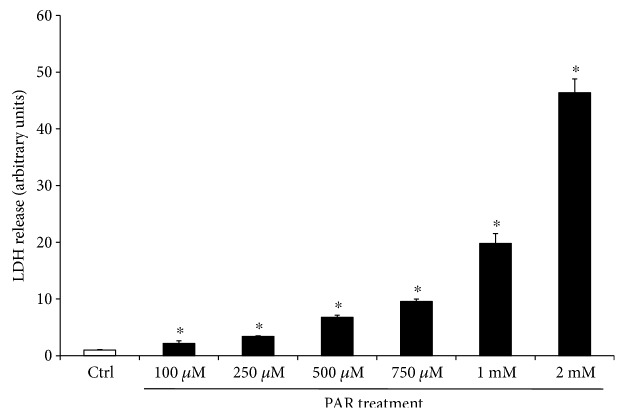
Cytotoxic effects of PAR treatment in A375 cells. Melanoma cells were exposed to different doses of PAR (100 *μ*M–2 mM) for 24 h. Cytotoxicity was calculated by measuring the amount of LDH released from the cytosol of PAR-damaged cells into the supernatant. Data are the average ± SD of three independent experiments. ^∗^*p* < 0.05 versus control.

**Figure 3:**
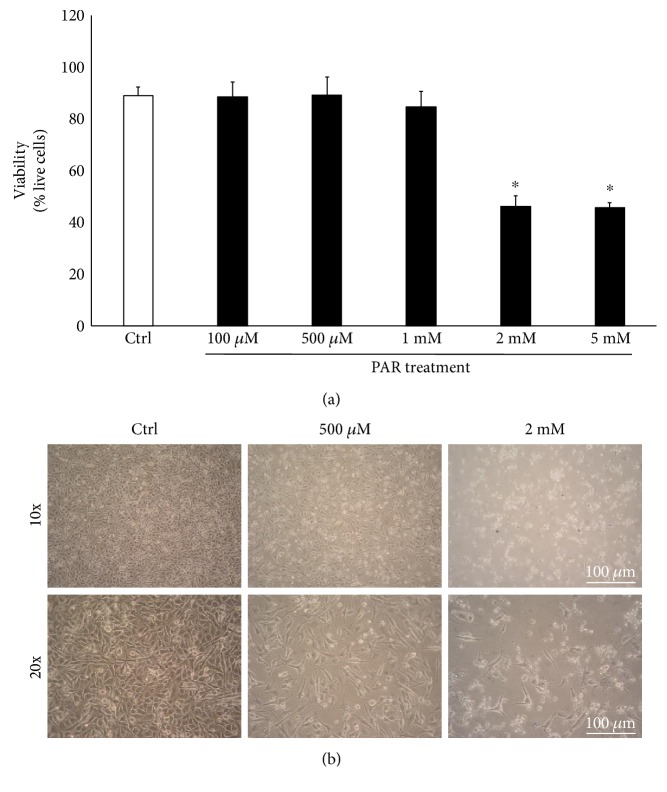
Effects of PAR treatment on A375 melanoma cell viability. (a) Melanoma cells were exposed to different doses of PAR (100 *μ*M–2 mM) for 24 h. Flow cytometric analysis demonstrated a significant decrease of cell viability at 2 mM dose of PAR. Data are the average ± SD of three independent experiments. (b) Results of cell viability were confirmed by visual assessment. Representative bright light microscopy images at 10x and 20x magnification of the optical microscope. ^∗^*p* < 0.05 versus control.

The first set of experiments evaluated the cytotoxicity induced by PAR on the A375 cell line. After 24 hrs of PAR treatment (from 100 *μ*M to 2 mM), the lactate dehydrogenase (LDH) release indicates a significant dose-dependent cytotoxicity (Figure 2), which is remarkable at PAR concentration of 2 mM dose (LDH release 35-fold higher than control cells). PAR cytotoxicity was confirmed by flow cytometry (Figure 3(a)) and optical microscope analysis (Figure 3(b)), showing a dose-dependent effect.

### 3.2. Effect of PAR on Melanoma Cell Proliferation

**Figure 4:**
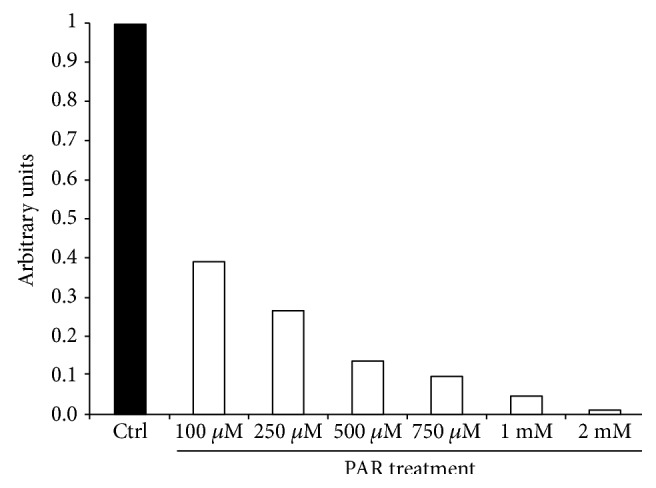
Effect of PAR treatment on A375 melanoma cell proliferation. The cell proliferation rate with respect to the alive cells after 24 hrs of treatment with PAR at different doses was determined. The proliferation was expressed as BrdU incorporation in A375 cells.

The results shown in Figure 4 indicate a dose-dependent decrease in cell proliferation compared to control cells. Based on these results and the data present in the literature [26], we have performed our next experiments at the doses of 500 *μ*M and 2 mM.

### 3.3. Effect of PAR on Connexin 43 Expression

**Figure 5:**
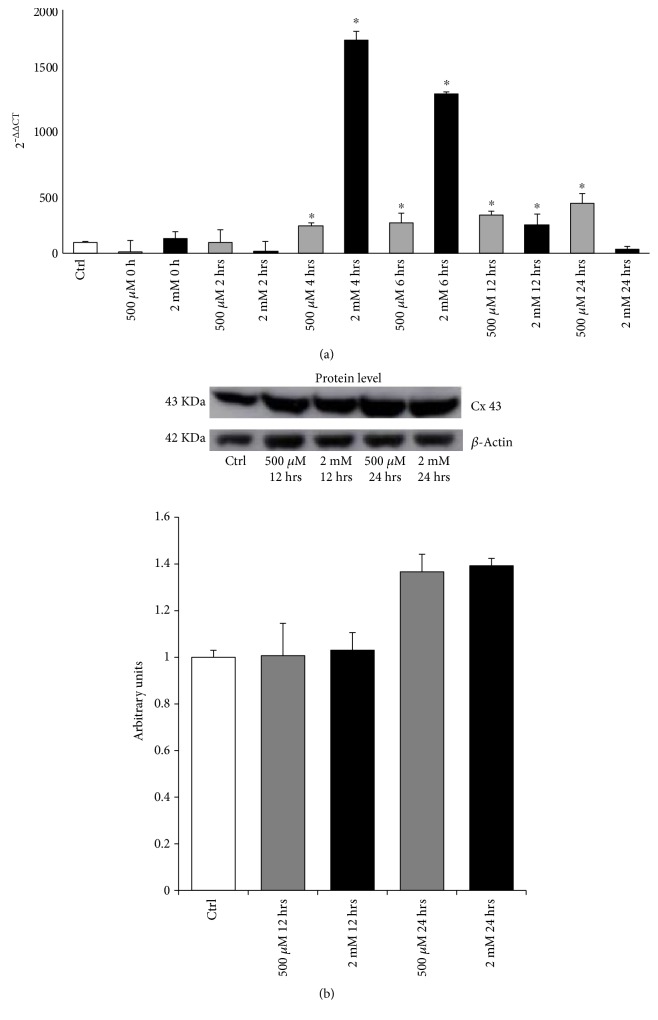
Effects of PAR treatment on the Connexin 43 levels in A375 melanoma cell. (a) Connexin 43 mRNA expression levels of control and cells treated with two different doses of PAR (500 *μ*M and 2 mM) at different time points. Transcript levels for Connexin 43 were evaluated by q-PCR analysis. Results are the means ± SD of three independent experiments, each analyzed in triplicate. (b) Representative Western blot for Connexin 43 is depicted in the top of panel and the quantification of the bands (average ± SD) in the bottom panel. Data are expressed in arbitrary units (averages of three experiments, ^∗^*p* < 0.05 versus control).

The gap junctions (GJs) are hemichannels, called connexons, each formed by six connexins. GJs allow intercellular communication and the direct exchange of ions, second messengers, and metabolites among neighboring cells. Recent experimental evidence have ascribed to GJs an important role in carcinogenesis, suggesting that connexins could be potential targets for cancer prevention and possibly chemotherapy [27]. Therefore, we analyzed the expression of Connexin 43 (CX43), the main component of the gap junctions, in melanoma cells after PAR treatment. As shown in Figure 5(a), PAR treatment significantly and time-dependently induced the expression of CX43 gene, starting at 4 hrs and further increasing up to 24 hrs for 500 *μ*M dose. A different trend was observed with 2 mM treatment, where the peak of CX43 expression is reached already at 4 hrs, followed by a progressive decrease, reaching the control level at 24 hrs (Figure 5(a)). The gene expression data correlate with the CX43 protein levels; indeed, Western blot analysis (Figure 5(b)) revealed that the increased level of CX43 protein is still maintained at 24 hrs time point.

### 3.4. Effect of PAR Treatment on Ultrastructure of Melanoma Spheroids

**Figure 6:**
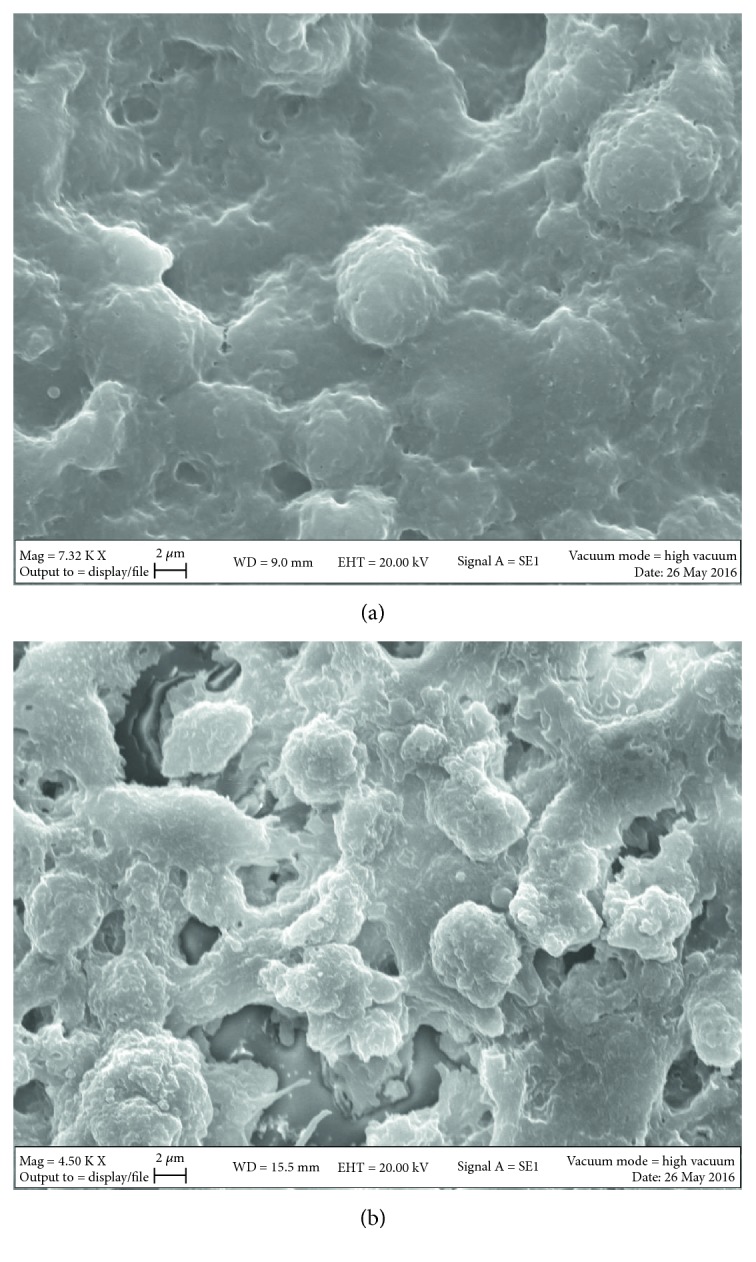
Scanning electron microscopy images of melanoma spheroids treated with PAR. Cell morphology of untreated spheroid ((a) magnification 7320x) and spheroid treated with 500 *μ*M dose of PAR for 28 days ((b) magnification 4500x), as visualized by scanning electron microscope (SEM).

To further confirm the effect of PAR on melanoma cells, we performed experiments on a 3D model, that is, on spheroids, generated with the hanging drop technique. A microscopic evaluation of melanoma spheroids was performed in order to evaluate more in detail the status of cells after PAR treatment. This assessment was made by scanning electron microscope (SEM). As shown in Figure 6(a), control spheroids showed a clear compact structure, with less cell-to-cell contact also due to the slightly bigger dimension of the cells; on the other hand, after treatment with 500 *μ*M of PAR (Figure 6(b)), this morphology was lost; cells looked smaller and there was an increase in the cell-to-cell contacts. Of note, it was impossible to evaluate the morphology of spheroids treated with 2 mM dose, because the treatment did not allow the formation of a compact 3D structure.

### 3.5. Effect of PAR on Gap Junctions

**Figure 7:**
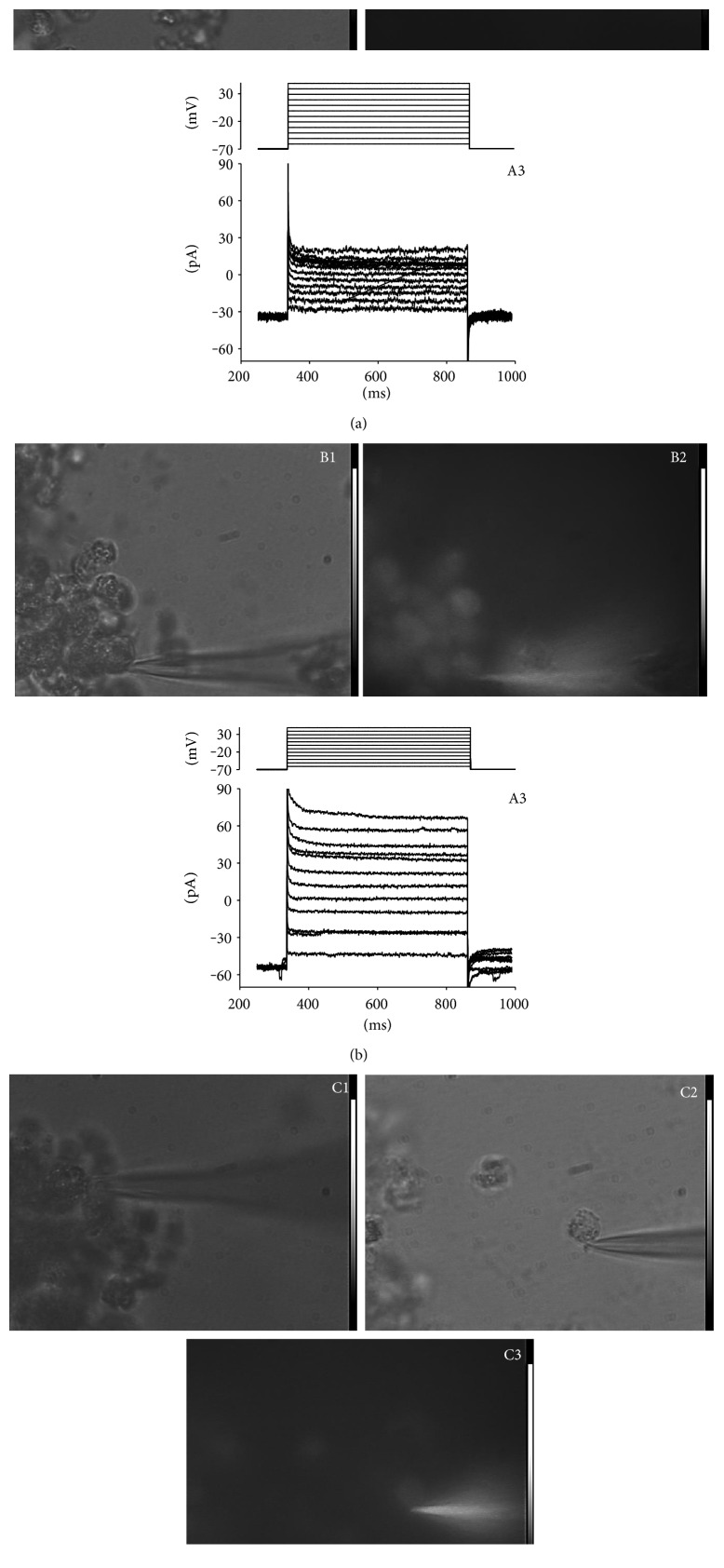
Effects of PAR treatment on intercellular communication in melanoma spheroids. Whole-cell and fluorescence imaging recording from cells untreated (a), treated with 500 *μ*M dose (b), and treated with 2 mM dose of PAR (c). Voltage protocol (traces in the upper portion of panel A3 and B3) consisted of 500 ms depolarizations from −60 to +50 mV in 10 mV steps, starting from the holding potential of −70 mV. Whole-cell, voltage-clamped currents (noisy traces in the lower portion of panel A3 and B3) were low-pass filtered at 500 Hz.

To confirm the increased level of gap junctions evidenced in the 2D experiments, cell-to-cell communications were assessed by injecting the fluorescent dye, lucifer yellow (LY), in the 3D model after PAR treatment (Figure 7). In control cells (A2), there was a negligible diffusion of LY, even after 20 min of recording (the corresponding bright field image is shown in Figure 1(a)), and cells exhibited high membrane resistance (>1 G*Ω*; the cell visualized in Figure 7(a) had a resistance of 1.4 GΩ). Moreover, there was a lack of any voltage- or time-dependent channels (Figure 7A3; recorded from the same cell). Conversely, in A375 cells treated with 500 *μ*M PAR, fluorescent dye diffused out of the cell toward the neighbor cells (Figure 7(B2); picture taken after 5 minutes of whole-cell recording; Figure 7(B1) is the corresponding bright field image) and the membrane resistance was much smaller (<0.6 GΩ; the cell of Figure 6(b) had a resistance of 0.59 GΩ), showing that this cell was electrically connected to the neighboring ones. Again, no sign of any voltage- or time-dependent channels was detected (Figure 7(B3); recording from the same cell). Cells treated with 2 mM PAR were severely damaged and acquired the typical “ghost”-like morphology (Figure 7(C1 and C2)). It was also possible to disengage the recorded cell from the cluster (Figure 7(C2)) by moving the patch pipette away from the cluster, indicating that the formation of a 3D compact structure was compromised at the highest dose. In this condition, the dye diffused out of the cell through the damaged membrane (Figure 7(C3)) and therefore gave a fainter fluorescence in comparison to Figure 7(A2 and B2). Consistently, the membrane resistance was very low (<100 MΩ).

### 3.6. Effect of PAR Treatment on Spheroid Size

**Figure 8:**
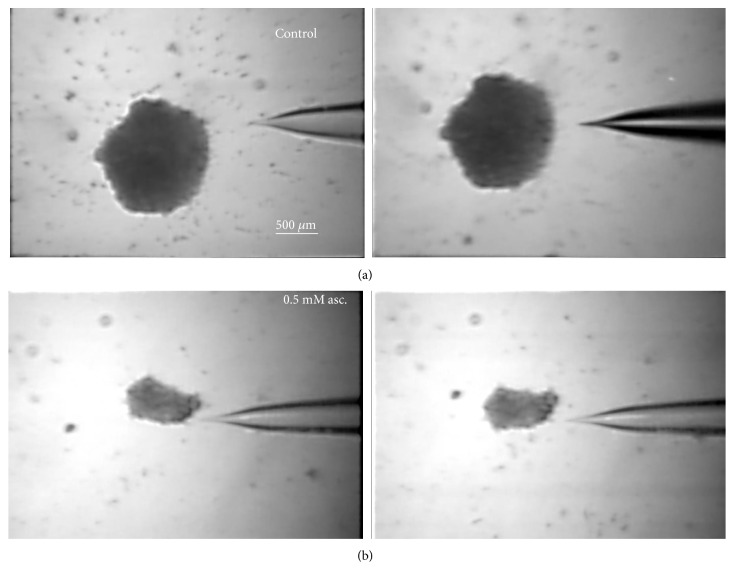
Effects of PAR treatment on the sizes of melanoma spheroids. Difference of sizes between control spheroids (a) and spheroids treated with 500 *μ*M of PAR for 28 days (b). Magnification 40x.

The volume of control spheroids was 2.83 ± 0.50 × 10^7^ *μ*m^3^, while the spheroids treated with 500 *μ*M PAR had a volume of 0.95 ± 0.27 × 10^7^ *μ*m^3^. Therefore, 500 *μ*M PAR treatment resulted in a threefold reduction of spheroid volume (Figure 8), consistently with the decrease of cell proliferation and viability, as determined in the previous experiments in 2D.

## 4. Discussion

Melanoma is the most dangerous form of skin cancer. Among Caucasian people, its incidence and related deaths are increasing yearly and more rapidly than other solid tumors. Dacarbazine is usually the first line of treatment for melanoma patients until their BRAF status is known. Nowadays, the standard therapies can be bounded by the unique recognized adjuvant therapy: interferon-*α* (IFN-*α*) [9]. Thus, our goal was to evaluate the possible role of PAR as a new adjuvant therapy for melanoma treatment.

Concerning ascorbic acid (AA), since the ‘70s, there has been a controversy on its use against cancer. Cameron and Pauling showed that high doses of intravenously injected vitamin C increased 20-fold the average time of survival in advanced cancer patients [28, 29], but this study was denied by other researchers who obtained negative results [30, 31]. This dispute has lasted until recently, when Pauling's hypothesis was confirmed by a study published in 2015 by Yun et al., who demonstrated that vitamin C (sodium ascorbate) selectively kills KRAS and BRAF mutant colorectal cancer cells [32]. The hypothesized mechanism of action is based on the fact that AA, at concentrations higher than 1 mM, can cause a build-up of hydrogen peroxide (H_2_O_2_), which is preferentially toxic toward tumor cells [33]. However, PAR might have more potent and different effects than those elicited by AA, due to the presence of two other components, potassium and ribose, the synergic action of which allows the correction of the hypokalemic condition found in cancer cells [20].

Consistently with the synergic action of individual components, our results revealed that PAR started to be cytotoxic even at the dose of 100 *μ*M in melanoma cells and the cytotoxic response was dose-dependent (100 *μ*M–2 mM).

Cell proliferation is not the only altered aspect in cancer. The gap junction proteins, connexins, are important regulators of intercellular communication and cell growth, and mutations or loss of function of gap junctions have been found in a few diseases, including cancer. Dysregulation of connexin channels has been described to either enhance or suppress tumorigenesis and metastasis. For example, in melanoma, like in other cancers, gap junctions and connexins, specifically Connexin 43, are upregulated in invasive lesions and in cells that disseminate to the lymph nodes [34]. By contrast, a more recent study showed that the increased expression of Connexin 43 in melanoma suppresses cell proliferation and anchorage-independent growth and also reduces the size of melanoma when grown in an ex vivo system [35]. Therefore, the role of connexins in cancer and cancer cell dissemination is highly controversial and apparently difficult to be reconciled.

Nevertheless, the bulk of the literature reports a reduced gap junctional communication in many tumor types, as a result of either downregulated expression of connexins or their inability to form functional junctions. In a recent study, Tittarelli et al. reported that Connexin 43 downregulation induced an increased proliferation in four melanoma cell lines, while its overexpression reduced melanoma cell growth *in vivo* [36]. According to these results, the increase in Connexin 43 expression that we found after PAR treatment in the 2D model was associated to a significant inhibition of cell proliferation and viability. Such a growth inhibition by PAR was confirmed in the 3D model, where the two-dimensional size and volume of spheroids decreased. In addition, the increase in functional gap junctions by 500 *μ*M PAR, that we observed in the 3D structures by using the florescent dye lucifer yellow, was confirmed by the SEM analysis, showing that cells of treated spheroids appeared to be suffering and with abnormal intercellular contacts. In a clinical perspective, this result suggests that the PAR-induced increase in tumor compactness could potentially impair the metastatic potential and facilitate cancer mass removal by surgery.

We have shown that 2 mM PAR prevented the assembly of the 3D structures; leading to a significant percentage of dead cells in the 2D model, supporting the efficacy of this drug against melanoma cells. This result is extremely important, considering that normal cells are insensitive to 20 mM ascorbate [26].

Taken together, our results show the efficacy of PAR treatments against melanoma cells, according to data from literature where this adjuvant therapy has shown to exhibit beneficial effects *in vivo*, on precarcinogenic conditions, and *in vitro*, on tumor cell types different from melanoma cells. The view that PAR could be used as adjuvant compound (alternative to interferon-*α*) in melanoma therapy is also supported by the fact that it is a nontoxic compound, is easy to administer, has no short-term side effects, and is not expensive.

## Figures and Tables

**Table 1:** Primers sequencing for housekeeping and CX43 genes, with amplified size.

CX43	F: 5′-tcaagcctactcaactgctgg-3′ R: 5′-tgttacaacgaaaggcagactg-3′	60.1	125	96.5	39	GenBank Accession NM_000165
RPL13A	F: 5′-cccgtccggaacgtctataa-3′ R: 5′-ctagcgaaggctttgaaattcttc-3′	60.2	203	97.3	39	GenBank Accession NM 000977.2
RPL11A	F: 5′-tgcgggaacttcgcatccgc-3′ R: 5′-gggtctgccctgtgagctgc-3′	60.1	108	96.5	39	GenBank Accession NM 000975.2
GAPDH	F: 5′-tgacgctggggctggcattg-3 R: 5′-ggctggtggtccaggggtct-3′	60	134	94.6	39	GenBank Accession NM 002046.3
